# Data on using single- and mixed-mode resins for capture chromatography of recombinant human thioredoxin from Escherichia coli

**DOI:** 10.1016/j.dib.2020.106500

**Published:** 2020-11-06

**Authors:** Ayswarya Ravi, Emma Foster, Zivko Nikolov

**Affiliations:** 1 Department of Biological and Agricultural Engineering, Texas A&M University, USA; 2 Department of Biological and Agricultural Engineering and National Center for Therapeutics Manufacturing, Texas A&M University, USA

**Keywords:** High-throughput screening, Mixed-mode chromatography, Thioredoxin, Capture chromatography

## Abstract

This paper provides the data collected from screening chromatographic resins for their ability to bind and purify recombinant human thioredoxin from *Escherichia coli* lysate. This data was used by “Capture chromatography with mixed-mode resins: A case study with recombinant human thioredoxin from Escherichia coli” [1] to determine the optimal resin to use as a capture step to initiate downstream processing of thioredoxin. Five chromatography resins were screened using a 96-well filter plate to experiment on a wide range of pH and conductivity conditions in a shorter amount of time while saving on materials. Thioredoxin-producing *E. coli* was cultivated, harvested, and lysed according to Ravi et al [1]. Thioredoxin containing lysate was dialyzed into the binding conditions, pH from 5.0 to 9.0 and conductivity from 2.0 to 10.0 mS, applied to each resin and incubated with shaking for 0.5 h. Data gathered after the incubation period consisted of host cell protein and thioredoxin concentrations remaining in the supernatant, which was considered flowthrough for the remainder of this study. Samples containing high concentrations of thioredoxin after the experimental period indicate that thioredoxin did not bind to the resin at those conditions and should not be utilized as a capture step. Additionally, samples that contain low concentrations of host-cell proteins after the experimental period indicate large amounts of host-cell proteins bound to the resin. The corresponding conditions may not contribute to higher purity. Operating all screening experiments at small volumes allows for selecting optimal binding conditions while minimizing the burden on upfront biomass production.

## Specifications Table

**Table:** 

Subject	Filtration and Separation
Specific subject area	Capture of target protein from complex lysate utilizing a variety of chromatography resins
Type of data	Figure Table Code
How data were acquired	Cell-free lysate containing thioredoxin was applied to commercial resins at specific pH and conductivity values. pH was measured using Thermo Scientific Orion Star A122 and conductivity by VWR conductivity meter. Flow through was collected and host cell protein (HCP) and thioredoxin (hTrx) concentrations were analyzed by Bradford total protein assay and indirect ELISA protein assay, respectively. Both assays are colorimetric, and the data collected were absorbance values at 595 nm for Bradford and 450 nm for the ELISA from the VersaMax plate reader.
Data format	Raw Analyzed
Parameters for data collection	A variety of chromatographic resins from single- and mixed-modal chemistries were screened for their ability to bind thioredoxin from a crude *E. coli* lysate. Buffer pH and conductivity levels were altered to determine the most favorable binding conditions for thioredoxin. pH ranges varied from 5-9 and conductivity ranged between 2-10 mS/cm except on Phenyl Sepharose resin where ammonium sulfate concentration ranged from 1-3M. These values were selected based on their likelihood to maintain the integrity of the target protein as well as being within the recommended operating conditions for the resin.
Description of data collection	Assay was followed and read using spectrophotometer to measure colorimetric differences and compared to standard curve.
Data source location	Texas A&M University, College Station, Texas-77843, USA
Data accessibility	With the article
Related research article	A. Ravi, E. Foster, L. Perez, Z. Nikolov, Capture chromatography with mixed-mode resins: A case study with recombinant human thioredoxin from Escherichia coli, J. Chrom. A. 1625, (2020). https://doi.org/10.1016/j.chroma.2020.461327

## Value of the Data

• Mixed-mode resins are a newly developing class of chromatography resins that combine the separating power of multiple interaction chemistries. Several studies have shown these resins achieve separation during polishing of monoclonal antibody (mAb) purification processes [2, 3]. In this data, single- and mixed-mode resins are screened and utilized to capture recombinant human thioredoxin from a complex *E. coli* lysate.
• Researchers in academia, pharmaceutical, and diagnostic industries can make use of this data during process development.
• The binding data for three commercially available mixed-mode resins allows for comparison between thioredoxin and other target biologics. This dataset can also be useful to determine binding and/or elution screening conditions.
• By combining adsorption chemistries, a broader range of both pH and conductivity conditions can be utilized, reducing the number of operating steps thereby shortening the total processing time. Decreasing the number of processing steps required to purify a target protein is the goal when working with biologically derived products [4].

## 1 Data Description

**Fig. 1:**
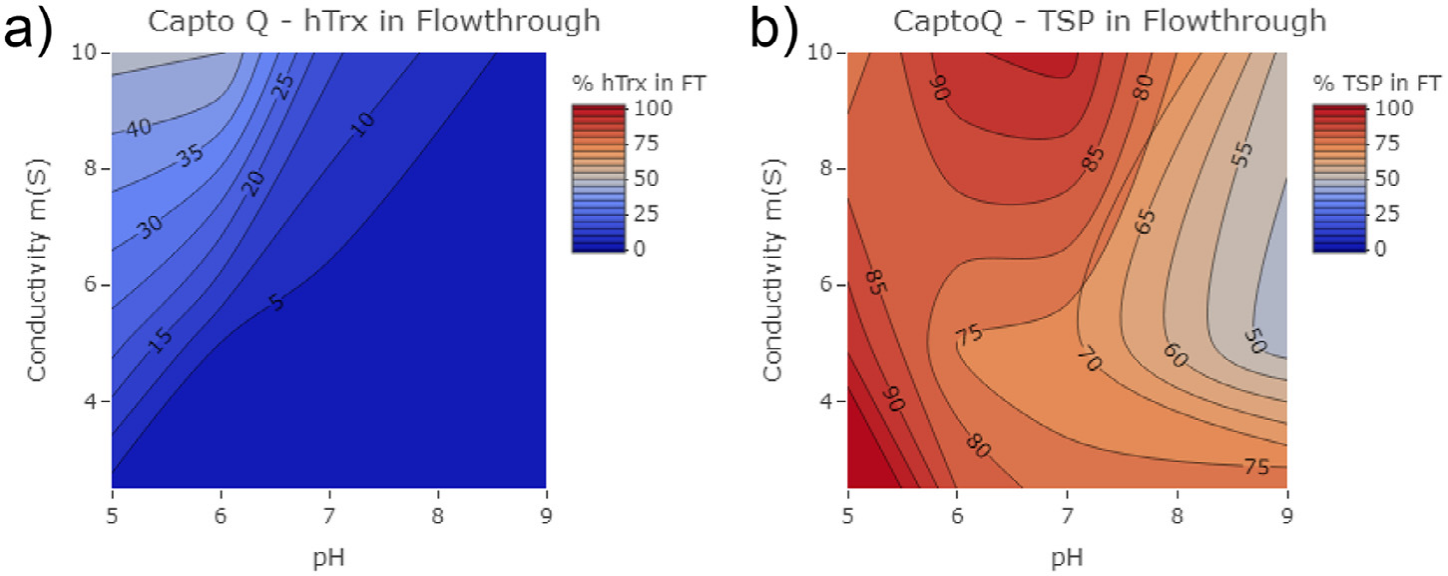
High-throughput screening of percent (a) hTrx and (b) HCP in Capto Q resin flow through across pH 5, 7 and 9 over 2–10 mS conductivity.

**Fig. 2:**
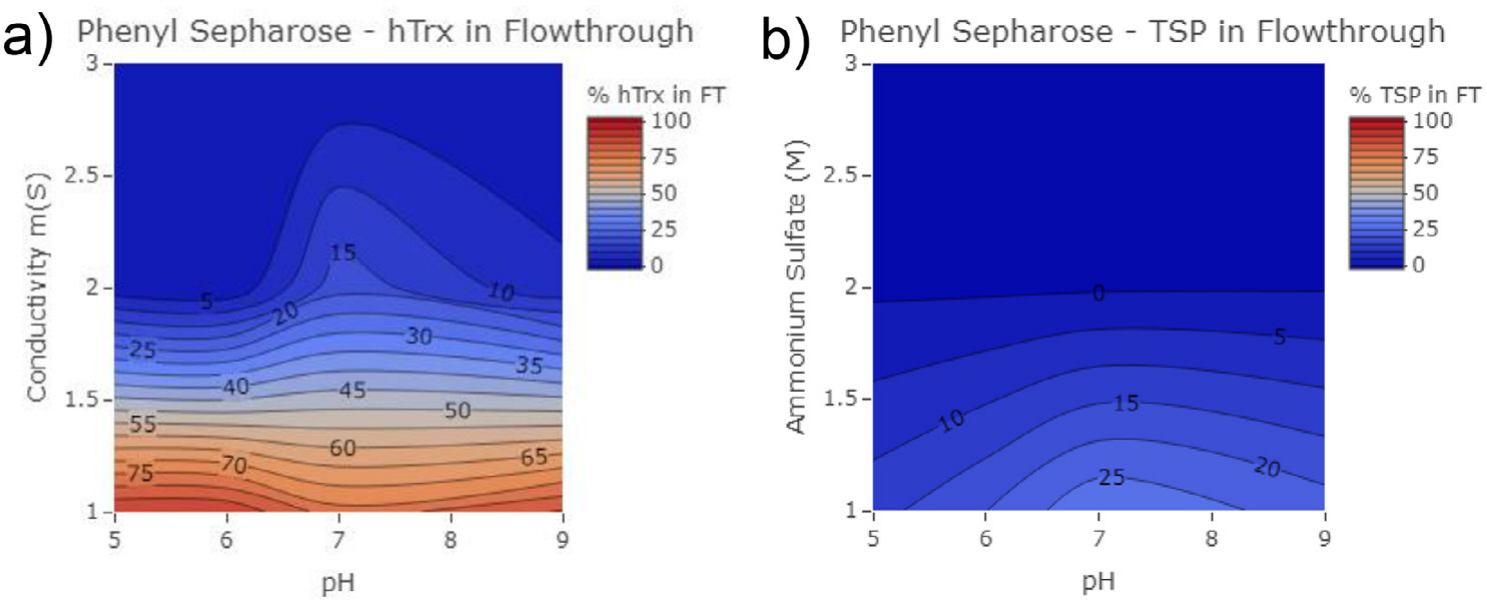
High-throughput screening of percent hTrx in Phenyl Sepharose resin flow through across pH 5, 7 and 9 over 1–3 M ammonium sulfate concentrations.

**Fig. 3:**
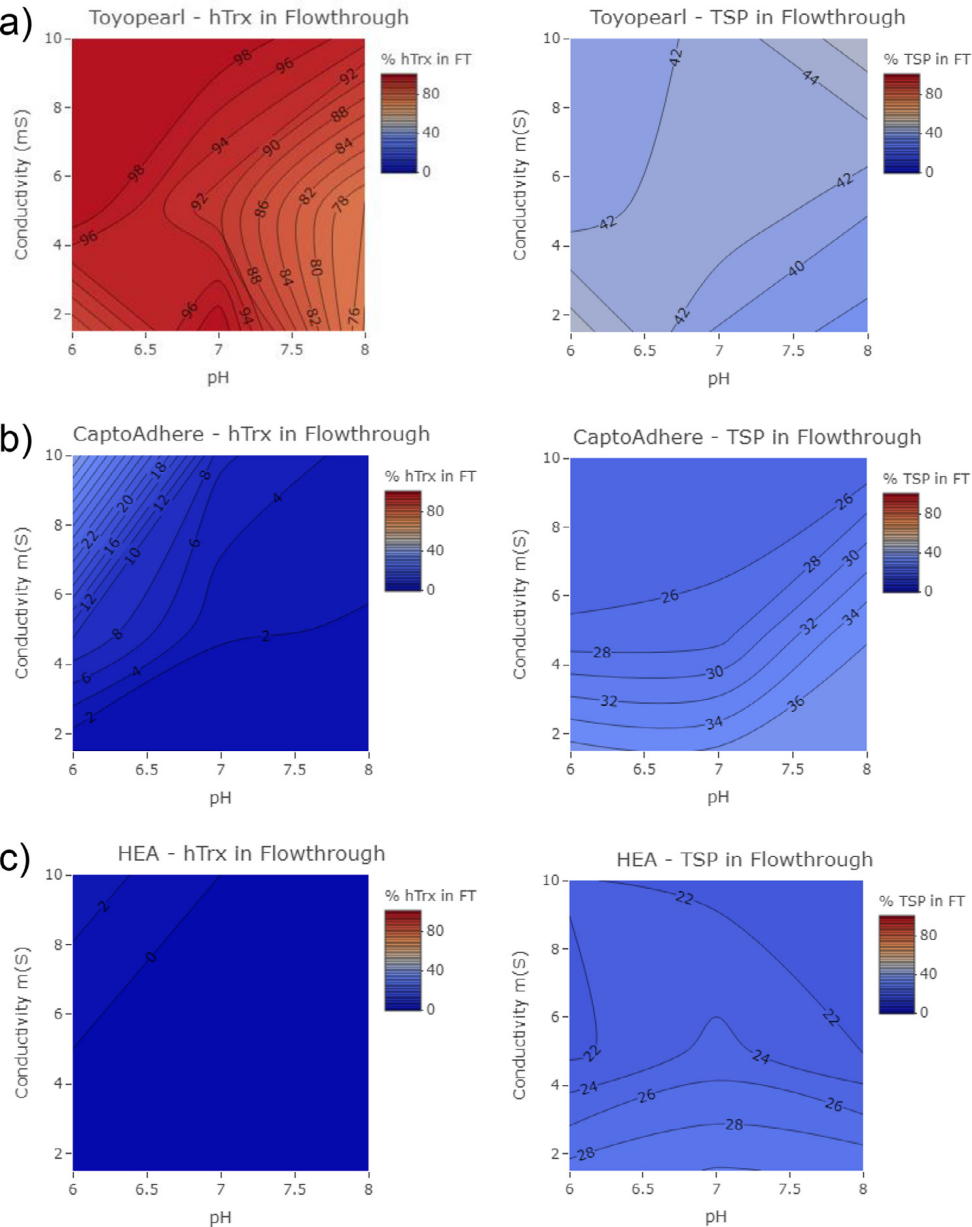
High-throughput screening of percent hTrx in flow through of (a) Toyopearl NH2-750F (b) Capto adhere and (c) HEA HyperCel mixed-mode resins across pH 6, 7 and 8 over 2–10 mS conductivities.

The data presented in this article are the result of high-throughput experiments conducted on a variety of chromatographic resins screened at varying pH and conductivity ranges. Fig. 1 is a set of contour plots displaying the trend for human thioredoxin (hTrx) and host-cell protein (HCP) capture on Capto Q resin. These plots were derived from the data file “CaptoQ_data.xlsx”. The same analysis was conducted on Phenyl Sepharose resin to create Fig. 2 from data file “PhenylSepharose_data.xlsx”. Fig. 3 shows additional contour plots resulting from analyzing pH and conductivity binding properties of hTrx to mixed-mode resins (a) Toyopearl NH2-750F, (b) Capto adhere, and (c) HEA HyperCel which were derived from data files “Toyopearl_data.xlsx”, “CaptoAdhere_data.xlsx”, and “HEA_data.xlsx” respectively. A relative binding ratio (k) defined as the ratio of the adsorbed hTrx (%) and HCP (%) was calculated for each pair of tested conditions for the mixed-mode resins to determine the adsorption preference of individual resins for hTrx and HCP as function of ionic strength and pH; these k values are shown in Table 1. The percent of adsorbed hTrx and HCP, was estimated by measuring the fraction of the initial protein, respectively, in the flow through.

**Table 1:** Relative binding ratio (k) for mixed-mode resins Toyopearl NH2 750F, Capto adhere and HEA HyperCel across pH 6, 7 and 8 over 2–10 mS conductivities. Data are averages of triplicate measurements.

	Toyopearl NH2 750F			Capto adhere			HEA HyperCel		
	2 mS	5 mS	10 mS	2 mS	5 mS	10 mS	2 mS	5 mS	10 mS
**pH 6**	0.27 ± 0.14	0.00 ± 0.00	0.05 ± 0.09	1.59 ± 0.09	1.32 ± 0.02	1.23 ± 0.07	1.41 ± 0.04	1.28 ± 0.07	1.26 ± 0.02
**pH 7**	0.03 ± 0.05	0.19 ± 0.33	0.01 ± 0.01	1.51 ± 0.02	1.33 ± 0.01	1.25 ± 0.01	1.43 ± 0.03	1.33 ± 0.02	1.27 ± 0.03
**pH 8**	0.41 ± 0.37	0.42 ± 0.40	0.19 ± 0.17	1.56 ± 0.03	1.33 ± 0.00	1.28 ± 0.01	1.43 ± 0.10	1.28 ± 0.04	1.27 ± 0.06

## 2 Experimental Design, Materials and Methods

### 2.1 Recombinant strain

Recombinant human thioredoxin (accession P105990) was expressed in BL-21 strain of *E. coli* which was provided by OrPro Therapeutics, Inc, California. The recombinant *E. coli* strain was engineered with a rhamnose-inducible promoter and kanamycin as the selection marker. *E. coli* culture was grown at 37 °C for 5 h in a 4 L New Brunswick bioreactor provided at the National Center for Therapeutics Manufacturing, Texas A&M University. Following initial growth stage, cells were induced with 5 mM rhamnose and culturing temperature was decreased to 32 °C for 6 hours. Both pH and dissolved oxygen were monitored during cultivation and were maintained at 7.0 and 40% respectively. Cell biomass was harvested by a refrigerated centrifuge at 4 °C. Maximal biomass recovery was achieved by using relative centrifugal force (RCF) of 15,000xg for 30 min and harvested cells were stored at –80 °C until use.

### 2.2 Lysate preparation

To maximize release of thioredoxin and reduce the release of contaminating host cell proteins (HCP) [1], a 50 mM sodium acetate, pH 5 buffer containing 5 mM DTT was used. To prepare lysates, one gram of frozen cells was resuspended in 10 mL of the lysis buffer before sonication. Resuspended cells were sonicated (QSonica 55 Watts, USA) in 15 mL polypropylene centrifuge tubes at 40% amplitude for 30 s on/off cycles for a total time of 5 min. To maintain the temperature of the lysate below 10 °C, the 15 mL centrifuge tubes were kept on ice during sonication. Cell debris was pelleted by centrifugation at 4 °C and RCF of 10,000xg for 30 min. The clarified lysate was collected and sterile filtered using a 0.2 µm PES syringe filter (Genesee Scientific, USA).

### 2.3 Analytical methods

Total protein concentrations were analyzed by Bradford protein assay (Thermo Fisher Scientific). Triplicate samples were diluted to be within the range of BSA standards (50–1500 µg/mL). Sample, standard, or blank was pipetted into 96-well plate (Corning) and incubated for 15 min at room temperature with Coomassie Protein Assay Reagent (Thermo Fisher Scientific, USA) before reading on VERSAMax microplate reader at 595 nm.

Thioredoxin concentrations were analyzed by indirect ELISA. Triplicate samples were diluted to be within the range of standards (0.1–3 µg/mL). Sample, standard, or blank was added to each well of a 96-well plate (Nunc maxisorb) and incubated overnight at 4 °C. All following incubation steps take place for 1.5 h at 37 °C shaking at 200 rpm on a Jitterbug microplate shaker. The plate with immobilized samples was washed with PBS-T, blocked with 1% BSA solution, and incubated. The plate was washed again, a 1:7500 dilution of anti-thioredoxin monoclonal antibody (Abcam, USA) was applied per well, and incubated. After washing the plate, a 1:10,000 dilution of anti-mouse polyclonal antibody conjugated with HRP (Sigma, USA) was applied per well, and incubated. Plate was washed, TMB developer (Sigma, USA) was applied to each well, and allowed to react until color was seen in the lowest standard. The reaction was stopped with 1M HCl before reading at 450 nm on VERSAMax plate reader.

### 2.4 Resin screening

Resin screening occurred by equilibrating each resin in the proper binding buffer corresponding to the condition to be tested. 50 µL of each resin was pipetted per well onto a 96-well filter plate from Empore and 250 µL of clarified, sterile filtered lysate adjusted to corresponding buffer conditions was applied to each well. All samples were run in triplicates. Binding of protein to resins occurred at room temperature for 30 min while vortexing. The flow through and resulting unbound protein was collected into receiving plate by centrifugation at 3000xg for 2 min or by applying a vacuum. The flow through was analyzed for HCP and thioredoxin content. For effective capture, hTrx in the flow through should be low and for an increase in purity, HCP in the flow through should be high.

#### 2.4.1 Single mode resins

Initially, single mode resins were screened for their ability to capture thioredoxin in a variety of binding conditions. CaptoQ, a single-mode anion exchange resin, and phenyl Sepharose, a single-mode hydrophobic interaction resin, were studied. Equilibrium binding of hTrx to Capto Q resin was conducted at pH 5, 7, and 9 and conductivities 2, 5, and 10 mS.

Equilibrium binding of hTrx to Phenyl Sepharose resin was conducted at pH 5, 7, and 9 and at ammonium sulfate concentrations ranging from 1 to 3 M.

#### 2.4.2 Mixed mode resins

After single chemistry resins showed mediocre capture and purity and narrow binding conditions, three mixed-mode resins; Toyopearl NH2-750F, CaptoAdhere, and HEA HyperCel, were screened. All were tested under the same binding condition ranges: pH 6, 7, and 8 and conductivity of 2–10 mS. Fig. 3 shows the hTrx and HCP content of the flow through for (a) Toyopearl NH2-750F, (b) CaptoAdhere, and (c) HEA HyperCel.

The binding values for Toyopearl, which range between 0.00 and 0.42 across the three pH and conductivity values tested, are consistent with poor binding of hTrx throughout the whole range of conditions tested (Fig. 3a). Compared to Toyopearl, Capto adhere had k-values greater than 1.0 in the whole range indicating an improvement in binding of hTrx as well as a decrease in the amount of HCP that adsorbed to the resin. The adsorption of hTrx to HEA HyperCel resin also had a strong salt tolerance at pH 6 to 8, and with consistently high k-values ranging from 1.26 to 1.43. Both Capto adhere and HEA binding ratios agree with Fig. 3b and c.

## Declaration of Competing Interest

The authors declare no known competing financial interest or personal relationships that could have or could be perceived to have influenced this report.

## Appendix Supplementary materials

Image, application 1

Image, application 2

Image, application 3

Image, application 4

Image, application 5
